# Quantitative analysis of Tr1 lymphocytes in patients with type 2 diabetes mellitus

**DOI:** 10.1007/s40618-023-02250-w

**Published:** 2024-01-06

**Authors:** C. Knott-Torcal, N. S. de la Blanca, A. Serrano-Somavilla, R. M. Hernández, M. Sampedro-Núñez, B. Ruiz-Rosso, S. Jiménez-Blanco, R. González-Amaro, L. González-Baranda, A. Garcimartin, M. Marazuela

**Affiliations:** 1 Department of Endocrinology and Nutrition, Health Research Institute, Hospital Universitario de La Princesa, Universidad Autónoma de Madrid, C/Diego de León 62, 28006 Madrid, Spain; 2 Faculty of Pharmacy, Universidad Complutense de Madrid, Av. Séneca, 2, 28040 Madrid, Spain; 3 Research Center of Health Sciences and Biomedicine (CICSaB), Universidad Autónoma de San Luis Potosí, SLP, México

**Keywords:** Tr1 lymphocytes, T2DM, T regulatory cells

## Abstract

**Background:**

Type 2 diabetes mellitus (T2DM) is usually accompanied by a low-grade inflammatory phenomenon, which participates in the pathogenesis of different complications of this condition. The inflammatory response is under the regulation of different mechanisms, including T regulatory (Treg) lymphocytes. However, the possible role of type 1 T regulatory (Tr1) cells in T2DM has not been explored so far.

**Aim:**

To carry out a quantitative analysis of Tr1 lymphocytes and other immune cell subsets in patients with T2DM and correlate these results with clinical findings and treatments.

**Materials and methods:**

Sixty patients with T2DM and twenty-three healthy controls were included in the study. Biochemical and anthropometric variables were evaluated, and Tr1 lymphocytes (CD4^+^CD49^+^LAG-3^+^IL-10^+^) and other cell subsets (Th17, Th22 and Foxp3 + Treg cells) were analyzed in peripheral blood samples by multiparametric flow cytometry.

**Results:**

Significant increased levels of Tr1 cells were detected in patients with severe and mild disease, compared to healthy controls. In addition, CD4^+^IL-10^+^ lymphocytes were also increased in patients with T2DM. In contrast, similar levels of Foxp3^+^ Treg cells, Th17 and Th22 lymphocytes were observed in patients and controls. Likewise, no significant associations were detected between Tr1 cell levels and different clinical and laboratory parameters. However, those patients receiving glucagon-like peptide-1 receptor agonists (GLP-1-RA) showed similar levels of Tr1 cells than healthy controls, and significant lower numbers than untreated patients.

**Conclusion:**

We observed an increase in Tr1 and CD4^+^IL10^+^ lymphocyte levels in T2DM. Moreover, GLP1-RA treatment was significantly associated with normalization of the Tr1 levels. This highlights another potential immune dysfunction in patients with T2DM, which could participate in the pathogenesis of this condition.

**Supplementary Information:**

The online version contains supplementary material available at 10.1007/s40618-023-02250-w.

## Introduction

Type 2 diabetes mellitus (T2DM) is a major challenge and an economic burden to public health in all countries [1]. This condition is characterised by the progressive appearance of different complications, which are tightly associated with the low-grade inflammatory phenomenon observed in these patients [2]. Although the pathogenesis of this inflammatory phenomenon has not been fully elucidated, the excess of adipose tissue and the increased production of proinflammatory cytokines, mainly tumor necrosis factor alpha (TNF-α) and interleukin-6 (IL-6), are clearly involved.

The activity of the immune system and the inflammatory response is under the regulation of different mechanisms, including the T regulatory (Treg) lymphocytes. In this regard, several subsets of these cells, including those described by Sakaguchi et al. [3], which are characterized by the phenotype CD4^+^CD25^high^Foxp3^+^, have been described. As expected, these regulatory lymphocytes have an important role in the inhibition of the immune response and inflammatory phenomena [4]. Accordingly, alterations in the number and/or function of Foxp3^+^ Treg cells have been described in patients with different autoimmune, allergic and inflammatory conditions [5]. Moreover, additional immune cells with regulatory activity have been described, including CD69^+^ Treg cells [6], as well as different subsets of B lymphocytes (Breg cells) [7].

Type 1 T regulatory (Tr1) cells are differentiated in the secondary lymphoid tissues and have an important role in promoting and maintaining the immune tolerance towards different antigens [8]. It has been described that these CD4^+^ T lymphocytes express high levels of the α2 chain of integrins (CD49b) as well as the LAG3 molecule (CD223), which is a structural and functional homologue of CD4 [9]. In contrast with the Treg cells described by Sakaguchi S, Tr1 lymphocytes do not show a constitutive expression of the transcription factor Foxp3 [10]. It has been described that these lymphocytes exert their immunoregulatory function through different mechanisms, including the release of the anti-inflammatory cytokine IL-10 [11]. As it has been reported, Tr1 cells exhibit the phenotype CD4^+^LAG3^+^CD49b^high^IL-10^+^Foxp3^−^, which allows their identification and quantification by flow cytometry [12].

As expected, several studies have analyzed the levels and the function of Foxp3^+^ Treg cells in T2DM. In most of them reduced levels of these cells have been detected in the peripheral blood of patients with this condition, which appears to contribute to the metabolic disturbances such as insulin resistance [13, 14]. In addition, other cells and cytokines involved in the inflammatory phenomenon have been analyzed in T2DM. In this regard, different studies have detected increased levels of Th17 cells in these patients. Accordingly, the Treg/Th17 ratio seems to be diminished in T2DM [15, 16], a phenomenon that is usually accompanied by increased serum levels of the pro-inflammatory cytokines TNF-α and IL-6 [17], and diminished concentrations of IL-10 [18]. However, at our best knowledge, no studies regarding the possible involvement of Tr1 cells in T2DM have been conducted.

A relevant factor in T2DM management is the possible effect of antidiabetic drugs on the immune system. Accordingly, the potential anti-inflammatory properties of newer antidiabetic drugs are being studied, mainly since the COVID-19 pandemic. In this regard, it has been reported that GLP-1-RA could exert an anti-inflammatory effect in patients with insulin resistance and psoriasis [19]. Furthermore, GPL-1 receptor signalling seems to be involved in the regulation of lymphocyte proliferation and maintenance of Treg cells as well as anti-inflammatory actions by inhibiting the activation of the transcription factor NF-κB [20–22]

The aim of this study was to carry out a quantitative analysis of Tr1 lymphocytes and other immune cells subsets in the peripheral blood from T2DM patients with severe and mild disease. Furthermore, we considered of interest to assess the possible effect of antidiabetic therapy on the immune parameters analyzed in this study.

## Materials and methods

### Patients

60 patients with T2DM were included in the study. Main clinical characteristics, including the body composition data, obtained through bioimpedance analysis are shown in Table 1. These patients were classified according to their disease severity in two cohorts. The first cohort (cohort 1) (*n* = 27, HbA1c > 7%, BMI ≥ 27) had evidence of a severe disease, with a high frequency of diabetes-related micro and macrovascular complications, whereas the second cohort (cohort 2) (*n* = 33, HbA1c > 5.6%, BMI ≥ 27) had a better metabolic control and a low frequency of diabetic complications. 62% of patients of the cohort 1 had diabetes-related complications, and all of them had at least one cardiovascular risk factor, such as hypertension or dyslipidemia, whereas in the cohort 2 only 21% of patients had diabetes complications and 39% of them had one cardiovascular risk factor. Regarding the metabolic syndrome (MetS) associated conditions, in cohort 1, 75% of patients had hypertension, and 75% dyslipidemia, whereas in cohort 2, 57.58% had hypertension and 61% an altered lipid profile (Table 1). Patients of the latter group were under therapy with one or two oral antidiabetic drugs (OAD).

**Table 1:** Characteristics of the study population

	Severe T2DM (n = 27)	Mild T2DM (n = 33)	*P*
Sex (M/F)	69/31%	49/51%	0.302
Age	66.4 ± 6.28	61.5 ± 8.6	**0.0271**
Hypertension	(12/16) 75%	(19/33) 57.58%	0.2354
Dyslipidaemia	(12/16) 75%	(20/33) 60.61%	0.3209
HT + dyslipidaemia	(8/16) 50%	(13/33) 39.39%	0.4817
Weight	88.19 ± 16	88.03 ± 13.2	0.973
BMI	32.28 ± 5.46	32.62 ± 3.52	0.821
% Fat mass	40.739 ± 6.114	42.268 ± 5.097	0.2726
% Fat free mass	59.270 ± 6.114	57.732 ± 5.097	0.2726
% Muscle mass	26.918 ± 5.006	25.276 ± 4.504	0.1806
% Total body water	44.447 ± 4.882	42.513 ± 4.376	0.09905
HbA1c (%)	7.6 ± 1.04	6.49 ± 0.91	**0.0015**
Glucose (mg/dL)	151.13 ± 35.25	129.5 ± 30.87	**0.0084**
Cholesterol (mg/dL)	167.63 ± 31.95	172.84 ± 36.92	0.616
Triglycerides (mg/dL)	144.81 ± 76.51	134.94 ± 57.31	1
T2DM macrovascular complications	(4/16) 25%	(4/33) 12.12%	0.413
Nephropathy	(4/16) 25%	(2/33) 6.06%	0.086
Retinopathy	(2/16) 12.5%	(1/33) 3.03%	0.245

Statistically significant *P* values are in bold

Data correspond to percentages and the arithmetic mean and SD

*T2DM* Type 2 diabetes mellitus, *HT* arterial hypertension, *BMI* body mass index, *HbA1c* glycosylated haemoglobin

Comparing both populations, the cohort 1 of patients (*n* = 16) was slightly older, with longer evolution of their T2DM, higher fasting glucose concentration and HbA1c than patients of cohort 2 (*p* = 0.0083 and *p* = 0.0015, respectively). Regarding anthropometric variables, no statistical differences were found between the two cohorts.

A control group of individuals, apparently healthy and without evidence of T2DM or MetS, was also included (*n* = 23), BMI of 26.2 ± 6.3 kg/m^2^, blood glucose levels of 86.7 ± 10.9 mg/dl and triglycerides levels of 64.4 ± 11.6 mg/dl).

This study was approved by the Internal Ethical Review Committee of the University Hospital La Princesa, and a written informed consent was signed by all the participants prior to their inclusion in the study, in accordance with the Declaration of Helsinki.

### Cells

Blood samples were drawn at University Hospital La Princesa to analyze routine biochemical parameters and to isolate peripheral blood mononuclear cells (PBMC), by density-gradient centrifugation with Ficoll–Paque (Lonza Ibérica S.A.U., Barcelona, Spain). Cellular viability was assessed by trypan blue dye exclusion, and it was always higher than 95%.

### Flow cytometry analysis

PBMCs were incubated for 30 min at 4 °C with the following monoclonal antibodies (mAb): anti-CD4-PerCp (BD Biosciences), anti-CD49b-APC (BioLegend), anti-CD25-APC (Miltenyi Biotec) and anti-LAG-3 (CD223)-Fluor450 (Thermo Fisher Scientific). Then, cells were fixed and permeabilized with the Foxp3 Fix/Perm kit (eBioscience) and treated with PFA 4% with saponine 0.1%, and cells were additionally stained with an anti-IL-10 PE ((BioLegend) or an anti-IL-22-APC (eBioscience) or an anti-IL-17A-APC/Cyanine7 (eBioscience) and an anti-Foxp3-FITC (Thermo Fisher Scientific) mAb. Finally, cells were analyzed in a FACSCanto flow cytometer (Becton–Dickinson), by using the FlowJo software v7.6 (Tree Star Inc, Ashland, OR). Results are presented as the percentages (relative values) of positive cells, which are related to the total lymphocyte number acquired per volume of the sample; or as the absolute values of Tr1 cells, normalized by total lymphocytes/mm^3^ obtained from blood samples.

### Statistical analysis

Results were expressed as the arithmetic mean and standard error of the mean (SEM) or the median and interquartile range. Continuous variables were assessed using Spearman’s rho analysis, whereas categorical variables were assessed using Student’s t-test/Mann Whitney U or one-way ANOVA/Kruskal–Wallis. Results were considered significant when p value was < 0.05. All the statistical analysis was performed with R version 4.0.3

## Results

### Levels of Tr1 cells in patients with T2DM

**Fig. 1:**
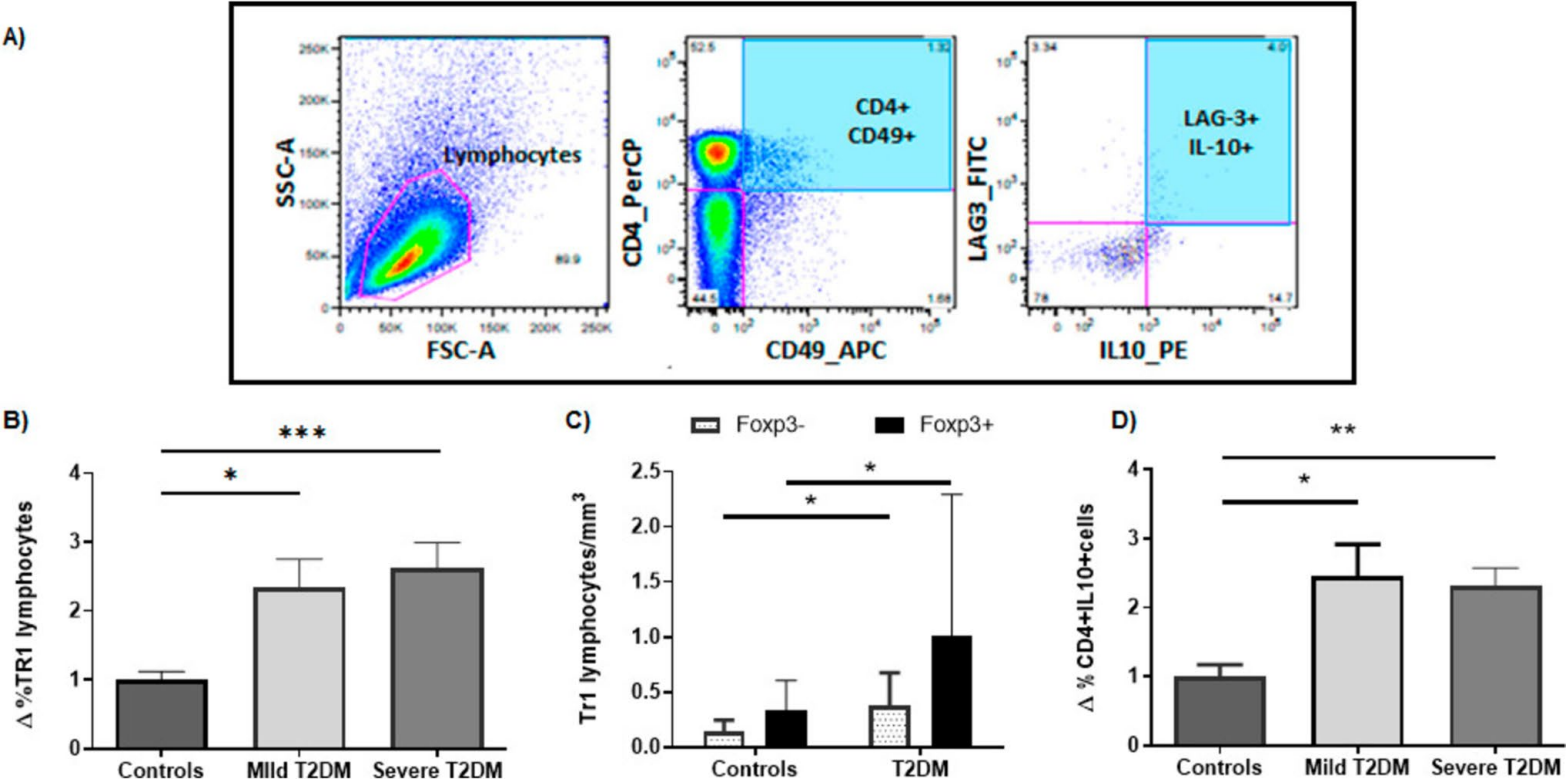
Quantitative analysis of Tr1 and CD4^+^IL-10^+^ lymphocytes in T2DM. Peripheral blood mononuclear cells from patients with T2DM and healthy controls were analyzed by flow cytometry for Tr1 cells (CD4^+^CD49^+^LAG-3^+^IL-10^+^) and CD4^+^IL-10^+^ lymphocytes, as stated in “Materials and Methods”. **A** Flow cytometry strategy for the analysis of CD4^+^CD49^+^LAG-3^+^IL-10^+^. Data of a representative healthy control are shown. **B** Fold increase of % positive/% control levels of Tr1 cells in the peripheral blood from healthy controls and patients with mild (and well-controlled) and severe disease. **C** Levels of Tr1 cells, expressing or not Foxp3 (black and grey bars, respectively) in the peripheral blood from healthy controls and patients with T2DM. **D** Proportion of CD4^+^IL-10^+^ lymphocytes in the peripheral blood from healthy controls and patients with mild and severe T2DM. Data correspond to the mean and SEM. *p < 0.05; ***p < 0.001

The flow cytometry strategy for the analysis of Tr1 lymphocytes is shown in Fig. 1A. When the levels of Tr1 lymphocytes (defined as CD4^+^CD49^+^LAG3^+^IL10^+^) were analyzed in both patients’ groups (severe and mild), we observed a significant fold increase in these cells in comparison with control individuals, either as percentage or absolute number of cells (Kruskal wallis test p = 0.0009; mean of controls = 1 ± 0.12, Mild T2DM = 2.3 ± 0.42 and Severe T2DM = 2.6 ± 0.37, p = 0.016 and p = 0.0003 respectively Fig. 1B, and data not shown). Since it has been described that some Tr1 cells can transiently upregulate FOXP3 upon activation of the Tr1 cells [10–12], we extended our flow cytometry analysis of these cells, including Foxp3 staining. As shown in Fig. 1C, the levels of both Tr1 cell subsets, expressing or not Foxp3, were significantly higher in T2DM patients compared to healthy controls (median controls = 0.13, T2DM = 0.28, p = 0.015 FOXP3−; median controls = 0.26, T2DM = 0.46, p = 0.04 FOXP3 +). Moreover, due to the potential relevance of the anti-inflammatory cytokine IL-10 in T2DM, we also analyzed the levels of T helper lymphocytes that synthesize this cytokine, and we have observed increased fold levels of CD4^+^IL-10^+^ cells in patients with mild and severe T2DM compared to healthy controls (Kruskal wallis test p = 0.0045; mean of controls = 1 ± 0.17, mild T2DM = 2.46 ± 0.46 and severe T2DM = 2.31 ± 1.76, p = 0.023 and p = 0.0013, respectively), Fig, 1D).

### Levels of Treg, Th17 and Th22 lymphocytes in patients with T2DM

**Fig. 2:**
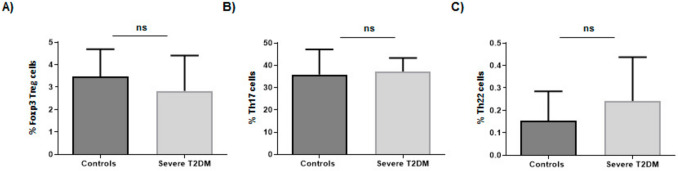
Quantitative analysis of different immune lymphocyte subsets in patients with T2DM. **A** Levels of Foxp3^+^ Treg cells in patients with severe T2DM and healthy controls. **B** Levels of Th17 lymphocytes in patients with severe T2DM and healthy controls. **C** Levels of Th22 lymphocytes in patients with severe T2DM and healthy controls. *ns* non-significant

As shown in Fig. 2A, similar levels of Foxp3^+^ Treg lymphocytes were observed in healthy controls and patients with T2DM (p > 0.05). In addition, no significant differences were observed in the levels of CD4^+^ cells synthesizing the pro-inflammatory cytokine IL-17 when patients and controls were compared (p > 0.05, Fig. 2B). Finally, the number of peripheral blood Th22 lymphocytes was also similar in T2DM patients and healthy controls (p > 0.05, Fig. 2C).

### Association analysis between clinical variables and Tr1 cell levels

To assess the possible association of antidiabetic therapy and levels of Tr1 and CD4^+^IL10^+^ cells, we performed an extensive correlation analysis with the different clinical and laboratory parameters of the second cohort of patients (mild T2DM). 24 patients (72.73%) were under metformin treatment, nine with iSGLT2 (27.27%), seven with iDPP4 (21.21%) and eight with GLP-1-RA (24.24%). As expected, most patients were receiving different combinations of these drugs, most of them with metformin (Supplementary Table 1).

**Fig. 3:**
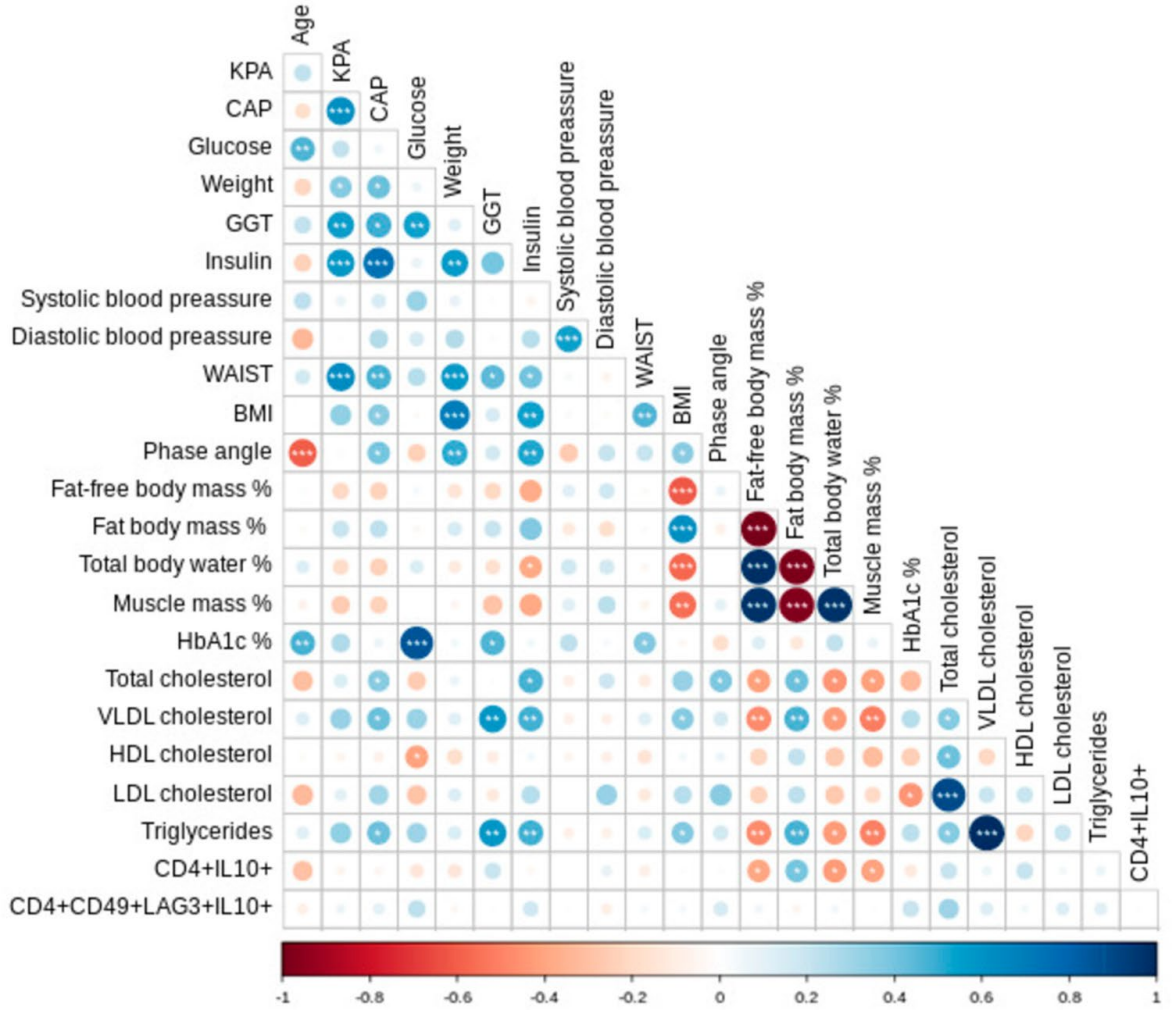
Association analysis of clinical, laboratory and immune cell subset parameters in patients with T2DM. A Spearman rho correlation heat map matrix is shown. Positive associations appear in blue, and negative correlations in red. As it is shown, the intensity of colors corresponds to the *r* value, and the level of significance is indicated with asterisks (*p < 0.05, **p < 0.01, ***p < 0.001). GGT, Gamma-Glutamyl Transferase; BMI, body mass index; HbAc1, glycosylated haemoglobin; VLDL cholesterol, very low-density lipoprotein cholesterol; HDL cholesterol, high density lipoprotein cholesterol; LDL cholesterol, low density lipoprotein cholesterol

Regarding CD4^+^IL-10^+^ cells, we observed a significant positive correlation with the percentage of fat body mass (r = 0.396, p = 0.028) as well as a negative association with the percentages of fat-free body mass and the muscle mass (r = −0.396, p = 0.028 and r = −0.401, p = 0.026; respectively, (Table 2; Fig. 3). In contrast, in the case of Tr1 cell levels, no apparent significant correlations were observed with any of the parameters analyzed (p > 0.05 in all cases, Fig. 3).

**Table 2:** Values of* r* and *p* of the correlation analyses between the levels of CD4^+^IL-10^+^ lymphocytes or Tr1 cell subsets and laboratory or bioimpedance analysis data from patients with T2DM

	CD4^+^IL10^+^		Tr1		Tr1 Foxp3^−^		Tr1 Foxp3^+^	
	*r*	*p*	*r*	*P*	*r*	*p*	*r*	*P*
% FFM	−0.39	0.028*	0.05	0.786	−0.11	0.556	−0.16	0.375
% FBM	0.39	0.028*	−0.05	0.786	0.11	0.556	0.16	0.375
% MM	−0.40	0.026*	0.01	0.935	−0.17	0.351	−0.20	0.267
% TBW	−0.42	0.018*	0.04	0.796	−0.13	0.464	−0.19	0.283

*FFM* fat free mass, *FBM* fat body mass, *MM* muscle mass, *TBW* Total body water

**Fig. 4:**
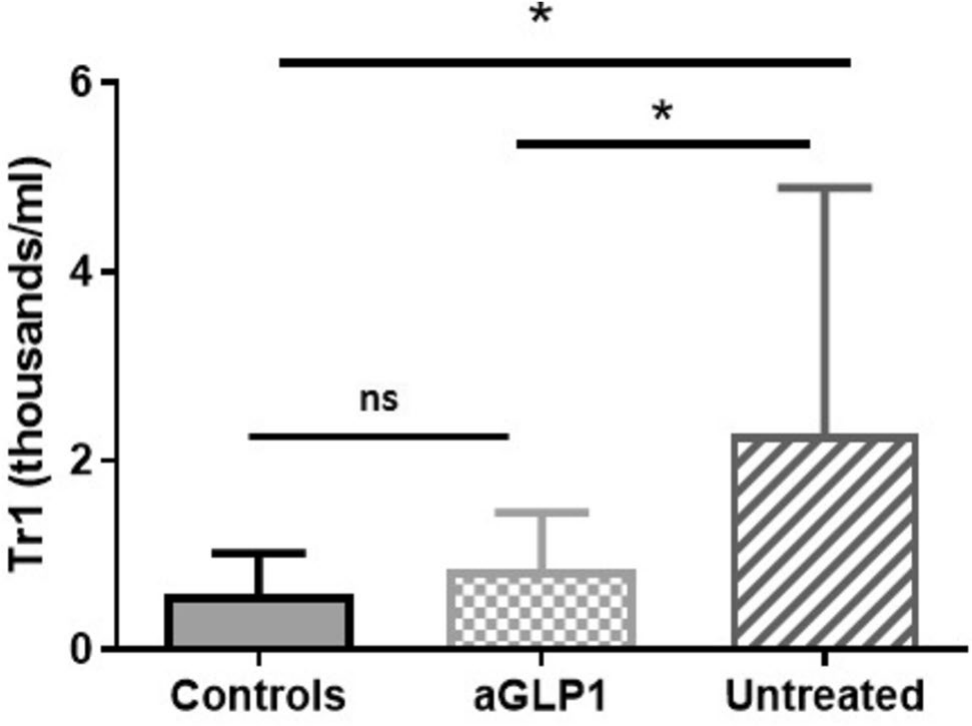
Levels of Tr1 cells and therapy with GLP-1-RA in patients with T2DM. Blood samples from healthy controls, T2DM patients, untreated and receiving glucagon-like peptide-1 receptor agonists (GLP-1-RA), were analyzed for the levels of Tr1 lymphocytes, as indicated in Materials and Methods. Data correspond to the median and Q3 quartile. ns, non-significant; *p < 0.05

Finally, an additional analysis showed than those patients included in the study that were under therapy with glucagon-like peptide-1 receptor agonists (GLP-1-RA) showed similar levels of Tr1 cells than healthy controls (p > 0.05, Fig. 4), and significant lower numbers than untreated patients (0.653 ± 0.726 vs 0.274 ± 0.193, p = 0.027; Fig. 4). In contrast, patients under metformin (alone or in combination therapy), iDDP4 and iSGLT2 did not show significant differences in Tr1 nor in CD4 + IL10 + levels (Supplementary Table 1).

## Discussion

In this study, we assessed the levels of different immune cell subsets, especially the Tr1 cell subset in the peripheral blood from different groups of patients with T2DM. Our results indicate an increase in the levels of Tr1 cells and CD4 + IL-10 + lymphocytes in T2DM, with no apparent abnormalities in the other lymphocyte subsets analysed. These abnormalities were not related to disease progression but to body mass composition. Interestingly, GLP-1-RA therapy but no other therapies were able to revert these immune abnormalities.

Low-grade inflammation is a well-known phenomenon that is commonly detected in patients with diabetes mellitus, and is clearly involved in different complications observed in this condition [2]. Moreover, different studies have analyzed the number and/or function of immunoregulatory cells in both, peripheral blood and fat tissue, from individuals with T2DM and/or obesity [4]. In this regard, it has been suggested that CD4^+^CD25^+^Foxp3^+^ Treg lymphocytes might have a relevant role in the pathogenesis of this inflammatory phenomenon [13, 14]. Likewise, the possible role of different subsets of regulatory B lymphocytes has also been explored in patients with T2DM and obesity [23, 24].

It has been established that Tr1 lymphocytes, as other immune regulatory cells, have an important role in promoting and maintaining the immune tolerance towards self and non-self-antigens [10]. Although the precise phenotype of Tr1 cells remained elusive by several years, it is now evident that these CD4^+^ T lymphocytes express high levels of the CD49b adhesion receptor along with the LAG3/CD223 molecule, showing a prominent capability to synthesize the anti-inflammatory cytokine IL-10 [25–27]. However, these regulatory lymphocytes do not show a constitutive expression of the transcription factor Foxp3 [10], a key phenotypic and functional characteristic of the Treg cells described by Sakaguchi et al. [28]. Therefore, Tr1 cells exhibit the phenotype CD4^+^LAG3^+^CD49b^high^IL-10^+^Foxp3^−^, which allows their identification and quantification by flow cytometry [12]. Nevertheless, it has been described that, as in the case of conventional T lymphocytes, Tr1 are able to express low-to-medium levels of Foxp3, with a functional role to be determined [10, 12]. Regulatory Tr1 cells seem to be involved in the pathogenesis of several immune-mediated disease, including type 1 diabetes mellitus, different inflammatory/autoimmune illnesses, and IgE-mediated hypersensitivity conditions [29].

In contrast to our hypothesis, we have detected that the T2DM patients included in this study showed increased levels of Tr1 cells. Several studies have detected significant enhanced numbers of other immune regulatory cells in patients with different conditions. Thus, patients with systemic lupus erythematosus show increased levels of CD69 + regulatory cells, with a diminished function of them [30], and similar findings have been reported in patients with active periodontal disease [30], an inflammatory condition that has been associated with other immune-mediated conditions, mainly rheumatoid arthritis. Furthermore, we have also observed increased numbers of different Treg cell subsets in the peripheral blood and thyroid tissue from patients with autoimmune thyroid disease [31]. Therefore, we consider that it is feasible that, under pathologic conditions, the defective function of a Treg cell subset would release a homeostatic signal that could induce the proliferation of these lymphocytes and their migration from the thymus to the peripheral blood. In this regard, it is worth mentioning that in this study we have not explored the function of Tr1 cells due to the limited number of cells available, hence this point remains as an interesting issue to be delved into.

One of the main characteristics of Tr1 lymphocytes is the synthesis and release of IL-10, a cytokine that has been widely considered as anti-inflammatory [32]. This Th2 cytokine has a relevant role in the activation and differentiation of B lymphocytes and the humoral immune response [33]. In addition, IL-10 exerts an important inhibitory effect on antigen presenting cells [34] and, therefore, on the activation and differentiation of T lymphocytes and the generation of the cellular immune response, which mediates many different chronic inflammatory phenomena [35]. Therefore, our results of increased levels of Tr1 cells and CD4^+^IL-10^+^ lymphocytes in T2DM are in agreement with previous reports regarding increased serum levels of IL-10 in patients with this condition as well as in prediabetic individuals and patients with metabolic syndrome [25]. In this regard, we found a direct correlation of CD4 + IL-10 + lymphocytes in T2DM and body composition parameters, with a direct relationship with the percentage of fat mass and an inverse correlation with muscle mass and fat free mass, pointing to an effect of body composition in these alterations. Although the possible consequences of all these immune aberrations requires additional studies, it is very feasible that they could contribute to the increased risk of T2DM patients for different infectious diseases.

Several studies have reported diminished levels of CD4 + CD25 + Foxp3 + Treg cells in the peripheral blood from patients with T2DM [14, 36]. Furthermore, Pitmon E, et al. recently observed that high glucose levels promote the differentiation of Treg lymphocytes [37]. Thus, we consider that our data on the normal levels of CD4 + CD25 + Foxp3 + Treg cells in the T2DM patients included in this study add valuable information to an interesting point that, to date, remains controversial.

As in the case of Treg cells, several studies have shown abnormal levels of Th17 cells in the peripheral blood from patients with T2DM [38]. Both increased and normal levels of CD4 + IL-17 + lymphocytes have been detected in these patients [16, 39, 40].

Th22 cells (CD4^+^IL-17^−^IL-22^+^) mainly exert their functions through the synthesis of IL-22, promoting antimicrobial immunity, tissue repair and inflammation [41]. Enhanced numbers of Th22 cell subset have been observed in different individuals with obesity and metabolically unhealthy [42, 43]. However, we have detected that both the levels of Th17 and Th22 cells are similar in T2DM and healthy controls suggesting that they do not have a relevant role in the pathogenesis of the inflammatory phenomenon observed in T2DM.

In recent years, there has been an increasing interest in the possible anti-inflammatory effects of antidiabetic drugs. In our study, we found significantly lower levels of Tr1 cells in patients treated with GLP-1-RA compared to those that had not received this treatment. Moreover, the possible protective effects of several antidiabetic drugs including GLP-1-RA has been explored in patients with COVID-19 [21, 44], as well as in patients with insulin resistance and psoriasis [19]. In addition, it has been reported that GLP-1-RA could exert a regulatory role on lymphocyte proliferation, the maintenance of Treg cells and the activation of transcription factor NF-κB [20–22].

We consider that one of the strengths of our study is the inclusion of two different cohorts of patients, well-matched in age, weight, and BMI, but one of them with a poorer metabolic control. However, the analysis of a larger number of individuals would strength the conclusions of our study and would allow a better definition of the possible effect of different antidiabetic drugs on immune parameters in T2DM.

## Conclusion

Our results indicate that the levels of peripheral blood Tr1 and CD4 + IL10 + cells are increased in patients with T2DM, at different stages of the disease, and that GLP-1-RA therapy might revert this immune dysregulation. Furthermore, body composition, rather than glucose metabolism, could play an important role in these immune alterations.

### Supplementary Information

Below is the link to the electronic supplementary material.

Supplementary file1 (DOCX 16 KB)

## Data Availability

Not applicable.
