# Supplementary material for: Quantitative analysis of Tr1 lymphocytes in patients with type 2 diabetes mellitus
Source: J Endocrinol Invest. 2024 Jan 6;47(6):1447–55. doi: 10.1007/s40618-023-02250-w (PMC11142976; doi:10.1007/s40618-023-02250-w)
Supplement: Supplementary file 1 — Supplementary file1 (DOCX 16 KB) [file 40618_2023_2250_MOESM1_ESM.docx]

**SUPPLEMENTARY TABLE.**

|  |  | **CD4+IL10+** | **CD4+CD49+LAG3+IL10+** | **CD4+CD49+LAG3+IL10+FOXP3-** | **CD4+CD49+LAG3+IL10+FOXP3+** |
| --- | --- | --- | --- | --- | --- |
| **Overall** |  | 84.939±100.252 | 0.558±0.653 | 0.19±0.245 | 0.339±0.362 |
| **Metformin** | No | 120.467±116.482 | 0.366±0.302 | 0.257±0.277 | 0.322±0.332 |
|  | Yes | 71.036±92.249 | 0.634±0.74 | 0.164±0.233 | 0.346±0.38 |
|  | p-value | 0.2760^b^ | 0.4831^b^ | 0.3313^b^ | 0.9812^b^ |
| **Metformin combination therapy** | No treatments | 229.098±178.671 | 0.366±0.302 | 0.257±0.277 | 0.322±0.332 |
|  | Only metformin | 325.578±223.461 | 0.981±1.196 | 0.318±0.419 | 0.535±0.508) |
|  | Metformin + 1 combination | 187.617±184.402 | 0.417±0.362 | 0.09±0.075 | 0.227±0.183 |
|  | Metformin + more than 1 combination | 237.52±233.405 | 0.616±0.621 | 0.132±0.1 | 0.339±0.428 |
|  | P-value | 0.6455 | 0.6731 | 0.5209 | 0.735 |
| **aGLP1** | No | 78.125±90.934 | 0.653±0.726 | 0.219±0.275 | 0.392±0.399 |
|  | Yes | 105.378±129.241 | 0.274±0.193 | 0.103±0.075 | 0.181±0.141 |
|  | p-value | 0.5076^b^ | **0.027^a^*** | 0.7169^b^ | **0.0344^a^*** |
| **iDPP4** | No | 86.637±105.74 | 0.566±0.703 | 0.19±0.258 | 0.352±0.39 |
|  | Yes | 77.579±79.551 | 0.528 ±0.42 | 0.191±0.195 | 0.284±0.22 |
|  | p-value | 0.9812000^b^ | 0.7242^b^ | 1.7242^b^ | 2.7242^b^ |
| **iSGLT2** | No | 97.948±108.12 | 0.574±0.712 | 0.225±0.273 | 0.377±0.403 |
|  | Yes | 45.911±61.47 | 0.512±0.471 | 0.084±0.055 | 0.227±0.166 |
|  | p-value | 0.2197^b^ | 1^b^ | 0.1883^b^ | 0.654^b^ |

**Supplementary table 1. Effect of antidiabetic drugs on the Tr1 populations.**

Values are expressed in mean±standard deviation. a=T-test, b=Mann-Whitney Wilcoxon. aGLP1= glucagon-like peptide-1 receptor agonist; iSGLT2= sodium-glucose co-transporter 2 inhibitors; iDPP4= dipeptidyl peptidase-4 inhibitors. p-value was considered significant when *p< 0.05
